# Efficacy of Transcutaneous Tibial Nerve Stimulation With Silver Spike Point® Electrodes for Refractory Overactive Bladder: A Single-Arm Study

**DOI:** 10.7759/cureus.34166

**Published:** 2023-01-24

**Authors:** Kanya Kaga, Tomonori Yamanishi, Tomohiko Kamasako, Mayuko Kaga, Miki Fuse, Mitsuru Ishizuka

**Affiliations:** 1 Urology, Chiba Prefectural Sawara Hospital, Katori, JPN; 2 Department of Urology, Continence Center, Dokkyo Medical University Hospital, Mibu, JPN; 3 Department of Urology, Utsunomiya Neurospinal Center Symphony Clinic, Utsunomiya, JPN; 4 Department of Urology, Saiseikai Utsunomiya Hospital, Utsunomiya, JPN; 5 Department of Urology, Mihama Narita Clinic, Narita, JPN; 6 Department of Urology, Yokohama Rosai Hospital, Yokohama, JPN; 7 Department of Gastroenterological Surgery, Dokkyo Medical University, Mibu, JPN

**Keywords:** ssp, ttns, tns, oab, silver spike point electrode, zhaohai point, sanyinjiao point, transcutaneous tibial nerve stimulation, tibial nerve stimulation, refractory overactive bladder

## Abstract

Background

Tibial nerve stimulation therapy is a treatment option for an overactive bladder. A surface electrode called a Silver Spike Point® electrode, which does not directly puncture the skin as in transcutaneous tibial nerve stimulation, but is expected to exert the same therapeutic effect as percutaneous tibial nerve stimulation, was developed. This study investigated the efficacy and safety of tibial nerve stimulation with Silver Spike Point® electrodes for refractory overactive bladder.

Methodology

This was a six-week, single-arm, prospective study on the efficacy and safety of transcutaneous tibial nerve stimulation for patients with refractory overactive bladder. Each treatment lasted 30 minutes and was performed twice a week. The stimulation sites of the tibial nerve were the Sanyinjiao point (SP6) and Zhaohai point (KI6) in both legs. The primary endpoint was the change in the total overactive bladder symptom score.

Results

In total, 29 patients (20 males and nine females: 64.86 ± 17.98 years old) were included in this study. Two women dropped out; one because of an adverse event and the other as requested. Therefore, 27 patients completed the study. The total overactive bladder symptom and International Consultation on Incontinence Questionnaire-Short Form scores significantly decreased by 2.22 and 2.39 points, respectively (p < 0.01 each). In the frequency volume chart, the numbers of urgency episodes and leaks in 24 hours significantly decreased by 1.53 and 0.44, respectively (p = 0.02 each).

Conclusions

Transcutaneous tibial nerve stimulation therapy using Silver Spike Point® electrodes was useful for patients with refractory overactive bladder and, thus, has potential as a new treatment option for refractory overactive bladder.

## Introduction

The efficacy of tibial nerve stimulation (TNS) for refractory overactive bladder (OAB) has been demonstrated [1,2]. TNS includes percutaneous tibial nerve stimulation (PTNS) with needle electrodes directly inserted into the posterior tibial nerve and transcutaneous tibial nerve stimulation (TTNS) using surface electrodes over the skin above the nerve. Currently, it remains unclear whether these therapies are equally effective; however, previous studies reported no significant difference in the efficacy of PTNS and TTNS for OAB [3,4]. Transcutaneous treatment is considered safe because it does not directly puncture the skin, whereas percutaneous treatment is more effective because it directly stimulates nerves in other practice areas [5]. Although electrical stimulation therapy using Silver Spike Point (SSP)® electrodes does not directly puncture the skin and is classified as transcutaneous therapy, its unique electrode shape stimulates deep into the skin with pinpoint accuracy, and similar outcomes to those with percutaneous therapy have been reported [6-8].

Therefore, the aim of the present study was to investigate the usefulness and safety of TTNS with SSP® electrodes for refractory OAB.

## Materials and methods

This was a single-arm, prospective study. All procedures were conducted according to the Declaration of Helsinki (UMIN000041660) and with the approval of the Institutional Review Board of Dokkyo Medical University (approval number: 29004). Signed informed consent was obtained from all patients before the initiation of treatment.

**Figure 1:**
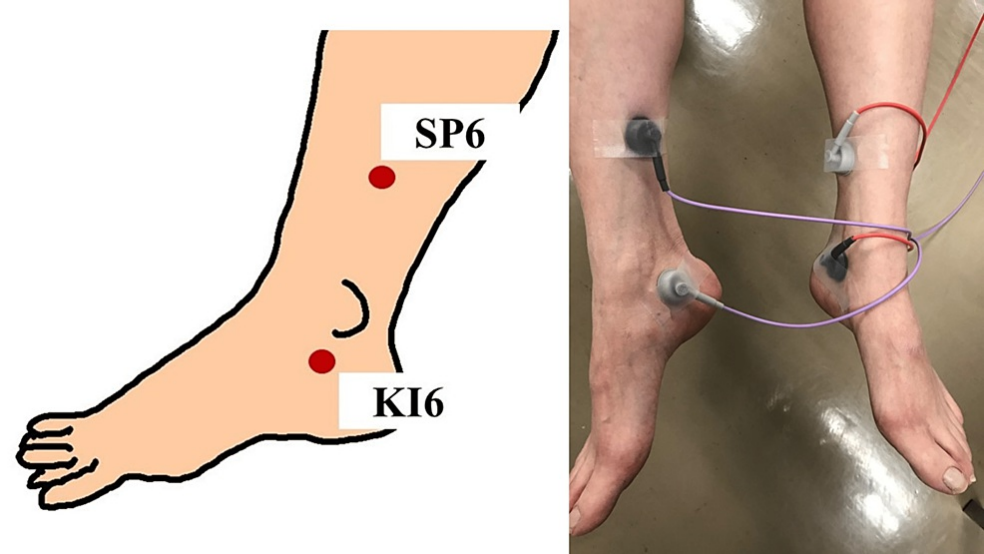
Stimulation points and image taken during transcutaneous tibial nerve stimulation. SP6: Sanyinjiao point; KI6: Zhaohai point

Patients with refractory OAB that had not been cured after more than three months of drug therapy were included. No age restrictions were imposed. Patients who were pregnant or possibly pregnant or who had a history of urinary tract infection, urinary tract stones, urethral disease, or pelvic surgery within 180 days prior to obtaining consent were excluded. Patients discontinued drug therapy for OAB, washed out for two weeks, and then received TTNS therapy twice a week for six weeks (12 times in total). One treatment was 30 minutes. In this study, we referred to the treatment protocol approved in Japan for magnetic stimulation therapy, a form of neuromodulation. It is envisioned to compare the therapeutic efficacy of this therapy with that of magnetic stimulation therapy in the future if efficacy is found in comparison with the sham group [9]. The stimulation sites of the tibial nerve were the Sanyinjiao point (SP6) and Zhaohai point (KI6) in both legs, and a surface electrode was placed at each site (Figure 1).

**Figure 2:**
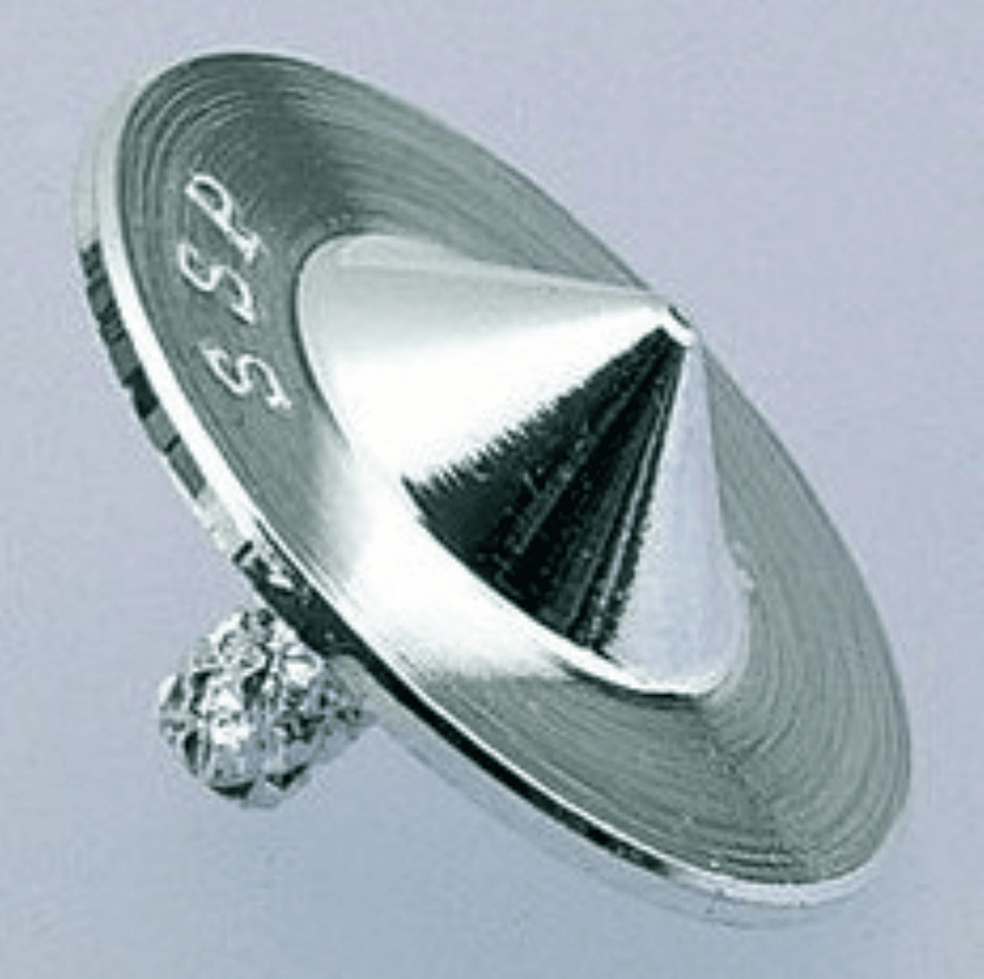
Silver Spike Point® electrode.

SP6 is located on three finger-breadths proximal to the medial malleolus, and KI6 is located on the depression below the medial malleolus [10,11]. Based on previous reports, the optimal stimulation conditions when using the electrodes described below were the low-frequency energization of bidirectional symmetric waves with a pulse width of 50 μs and a frequency of 100 Hz [12]. Surface electrodes and stimulators were SSP® electrodes and SSP Alpha 1® manufactured by Nihon Medix (Chiba, Japan) (Figure 2).

SSP® electrodes are metallic electrodes made of brass with silver plating. The tip of the SSP electrode adheres to the skin, deforming the stratum corneum and lowering its electrical resistance. As a result, the current is concentrated at the tip, enabling a point electrical stimulation.

The primary endpoint of this study was the change in the total OAB symptom score (OABSS). Secondary endpoints were subjective therapeutic effectiveness and changes in the International Consultation on Incontinence Questionnaire-Short Form (ICIQ-SF) score, free uroflowmetry, and frequency volume chart (FVC) before and after treatment.

Statistical analysis

The results obtained are shown as the mean ± SD. The Wilcoxon matched pair signed-rank test was performed to evaluate the significance of changes in parameters before and after treatment. The criterion for significance was p-values <0.05. The sample size was set according to the CONTACT study, the primary endpoint of which was total OABSS. A total of 26 patients were needed to yield 80% power to detect a difference of 2.00 points, assuming an SD of 2.50 points and α-error of 0.05 [13]. Therefore, 29 patients were required in consideration of a dropout rate of approximately 10%.

## Results

In total, 29 patients (20 males and nine females) were included in this study. The mean age was 64.86 ± 17.98 years (8-92 years). Seven patients had benign prostatic hyperplasia. Previous treatment included various drug therapies administered in the past, including at other hospitals, making it difficult to ascertain the details. Two women (6.90%) dropped out; one because of an adverse event, and the other as requested. Therefore, 27 patients completed the study. Results obtained before and after treatment are shown in Table 1 and Table 2.

**Table 1:** OABSS, ICIQ-SF, and FVC at baseline and week six. OABSS: overactive bladder symptom score; ICIQ-SF: International Consultation on Incontinence Questionnaire-Short Form; FVC: frequency volume chart

	Before	At six weeks	P-value
OABSS (n = 27)			
Total score, points	8.81 ± 2.80	6.59 ± 3.38	<0.01
Frequency score, points	1.04 ± 0.43	0.81 ± 0.47	0.01
Nocturia score, points	2.00 ± 0.86	1.70 ± 0.90	0.09
Urgency score, points	3.22 ± 1.42	2.26 ± 1.73	<0.01
Urgency incontinence score, points	2.56 ± 1.59	1.81 ± 1.68	0.02
ICIQ-SF (n = 23)			
Total score, points	8.91 ± 5.19	6.52 ± 5.63	<0.01
Frequency of leaks score, points	2.43 ± 1.61	1.87 ± 1.73	0.03
Amount of leaks score, points	2.26 ± 1.48	1.83 ± 1.55	0.06
Quality of life score, points	4.22 ± 2.87	2.83 ± 2.82	0.01
FVC (n = 21)			
Number of voids/daytime	8.94 ± 2.32	8.59 ± 2.32	0.33
Number of voids/night	1.63 ± 1.20	1.67 ± 0.98	0.83
Number of urgency episodes/24 hour	3.01 ± 3.64	1.48 ± 2.21	0.02
Number of leaks/24 hour	1.10 ± 1.63	0.66 ± 1.46	0.02
Amount of leaks/24 hour, mL	81.71 ± 223.17	79.71 ± 260.13	0.41
Total voided volume/daytime, mL	1,598.52 ± 536.53	1,576.49 ± 566.45	0.58
Total voided volume/night, mL	500.64 ± 262.24	545.79 ± 319.81	0.35
Nocturnal polyuria index, %	31.01 ± 11.62	33.48 ± 13.99	0.29
Mean voided volume, mL	153.94 ± 60.46	160.68 ± 62.52	0.45
Maximum voided volume, mL	273.81 ± 96.83	294.29 ± 162.79	0.34

**Table 2:** Urodynamic parameters at baseline and week six.

	Before	At six weeks	P-value
Free uroflowmetry (n = 10)			
Voided volume, mL	117.78 ± 44.74	113.37 ± 58.57	0.88
Mean flow rate, mL/s	7.62 ± 3.61	7.53 ± 3.03	0.72
Maximum flow rate, mL/s	12.17 ± 6.03	13.23 ± 5.16	0.96
Post-void residual, mL	30.30 ± 26.44	13.70 ± 17.02	0.16
Bladder voiding efficiency, %	85.21 ± 14.51	91.83 ± 8.54	0.50

The total OABSS and ICIQ-SF scores decreased at week six. Among OABSS subscores, decreases were observed in the urgency score and urgency incontinence score. Among ICIQ-SF subscores, the frequency of leak scores decreased, while the quality of life score improved. Regarding FVC, the number of urgency episodes and leaks in 24 hours significantly decreased. No significant differences were observed in free uroflowmetry.

## Discussion

Initially, PTNS was referred to as posterior TNS with needle insertion, that is, PTNS, and was synonymous with TNS. TTNS using surface electrodes without needles has recently been employed.

The exact mechanism of action of TNS is not known. Previous reports suggest that the mechanism of action is similar to sacral neuromodulation, which stimulates the S3 route. TNS ultimately modulates the reflex pathway by stimulating the posterior tibial nerve, a mixed nerve containing L4-S3 fibers [14].

Few studies have reported the use of electrical stimulation therapy using SSP® electrodes to treat urological diseases. Mallmann et al. compared parasacral TNS with TTNS using SSP® electrodes in women with OAB and demonstrated its usefulness [15]. However, its effects on refractory OAB were not examined. The present results demonstrated the efficacy and safety of TTNS with SSP® electrodes for refractory OAB. Total OABSS and frequency, urgency, and urgency incontinence scores significantly decreased. The total ICIQ-SF score and frequency of leaks score also significantly decreased, while the quality of life score significantly improved. Similar to the results obtained for OABSS and ICIQ-SF, the number of 24-hour urgency and leak episodes significantly decreased in FVC. Free flowmetry did not show any significant changes, indicating that TTNS did not affect dysuria. Furthermore, there was only one case of minor adverse events, such as pain, suggesting a high safety profile. SSP® electrodes have a unique conical shape and do not puncture the skin; therefore, their application does not cause pain. Stimulation therapy using SSP® electrodes has already been incorporated in the anesthesia and orthopedic fields. In the field of anesthesiology, acupuncture is more effective than surface electrodes for anesthetic effects, and anesthesia using SSP® electrodes can be considered a middle ground between acupuncture and treatment using surface electrodes [10]. To our knowledge, this is the first study to demonstrate the usefulness and safety of TTNS with SSP® electrodes for refractory OAB. Furthermore, because SSP® electrodes are reusable, the associated medical costs are cheaper than those of conventional disposable surface electrodes [16].

The male predominance of the patient population in this study needs to be considered. Previous studies on the efficacy of TNS, including TTNS, mostly involved female-only or female-dominant patient populations [2-4,15,17,18]. Therefore, although TNS therapy for OAB is already recommended in the guidelines, there have been few studies on a male-dominant population [19]. The present results demonstrated the efficacy and safety of TTNS for refractory OAB in a male-dominant patient population. International Prostatic Symptom Score voiding subscores, post-void residual urine volume (PVR), and bladder voiding efficiency were not affected by the treatment. Pharmacotherapy using anticholinergics or β3 agonists may cause voiding difficulties or increase PVR. Therefore, TTNS with SSP® electrodes may be safe for male patients with OAB.

The limitations of the present study include the absence of a control group because it was a single-arm, prospective study. Furthermore, the lack of studies with a male-dominant OAB patient population may be due to the difficulty of establishing patient backgrounds in male cases because confounding factors associated with comorbidities, such as benign prostatic hyperplasia (BPH), cannot be excluded. Furthermore, β3 agonists and anticholinergics, which are used in the treatment of OAB, and α1 blockers and phosphodiesterase 5 inhibitors, involved in the treatment of BPH, were withdrawn for two weeks before TTNS. Therefore, it was not possible to exclude all confounding factors. However, the present results suggest that TTNS was effective, even in male patients, because significant treatment efficacy was observed even in patients with conditions other than OAB.

Although the long-term effects have been reported for PTNS, the protocol for this study did not evaluate the long-term effects [1]. In addition, none of the literature in other areas evaluated the long-term effects.

## Conclusions

TTNS using SSP® electrodes is effective and safe for refractory OAB. Furthermore, TTNS therapy was useful for refractory OAB in a male-dominant patient population. Based on the present results, TTNS using SSP® has the potential as a new treatment option for OAB in men. Because this was a single-arm, prospective study, further accumulation of cases and a randomized controlled trial with a sham group are needed.
